# Closing the circle: current state and perspectives of circular RNA databases

**DOI:** 10.1093/bib/bbz175

**Published:** 2020-01-30

**Authors:** Marieke Vromman, Jo Vandesompele, Pieter-Jan Volders

**Affiliations:** 1 department of Biomolecular Medicine at Ghent University and a member of the Cancer Research Institute Ghent; 2 department of Biomolecular Medicine at Ghent University and a group leader at the Cancer Research Institute Ghent; 3 department of Biomolecular Medicine at Ghent University and at the Flemish Institute for Biotechnology, and a member of the Cancer Research Institute Ghent

**Keywords:** circRNA, circRNA databases

## Abstract

Circular RNAs (circRNAs) are covalently closed RNA molecules that have been linked to various diseases, including cancer. However, a precise function and working mechanism are lacking for the larger majority. Following many different experimental and computational approaches to identify circRNAs, multiple circRNA databases were developed as well. Unfortunately, there are several major issues with the current circRNA databases, which substantially hamper progression in the field. First, as the overlap in content is limited, a true reference set of circRNAs is lacking. This results from the low abundance and highly specific expression of circRNAs, and varying sequencing methods, data-analysis pipelines, and circRNA detection tools. A second major issue is the use of ambiguous nomenclature. Thus, redundant or even conflicting names for circRNAs across different databases contribute to the reproducibility crisis. Third, circRNA databases, in essence, rely on the position of the circRNA back-splice junction, whereas alternative splicing could result in circRNAs with different length and sequence. To uniquely identify a circRNA molecule, the full circular sequence is required. Fourth, circRNA databases annotate circRNAs’ microRNA binding and protein-coding potential, but these annotations are generally based on presumed circRNA sequences. Finally, several databases are not regularly updated, contain incomplete data or suffer from connectivity issues. In this review, we present a comprehensive overview of the current circRNA databases and their content, features, and usability. In addition to discussing the current issues regarding circRNA databases, we come with important suggestions to streamline further research in this growing field.

## Introduction

After their discovery more than three decades ago, circular RNAs (circRNAs) have been emerging as a large class of generally noncoding RNAs. Originating from the same precursor as linear RNA transcripts, circRNAs are formed through a process called back-splicing, in contrast to regular forward splicing. Back-splicing results in a covalently closed loop characterized by a nonlinear back-spliced junction (BSJ) between a splice donor and an upstream splice acceptor, and lacking a poly(A) tail and 5′ and 3′ ends. Due to their circular nature, circRNAs are more resistant to degradation by exonucleases and therefore, more stable than linear RNA [1,2]. CircRNAs are widespread and abundant in a variety of organisms. It is estimated that the total number of circRNA molecules is roughly 1% of the number of poly(A) molecules [3]. Generally, the expression levels of most circRNAs is estimated to be 5–10% of their corresponding linear RNA product [4]. Interestingly, the majority of circRNAs seem to be cell-type specific [3,5].

Although the function of most circRNA remains largely unknown, increasing evidence shows that circRNAs can act as a sponge for microRNAs (miRNA) and RNA binding proteins (RBPs), as modulator of transcription and splicing, and as template for translation [6,7]. Furthermore, circRNAs have been associated with a broad range of diseases, including various types of cancer [8,9]. Here, circRNAs have been found to act as miRNA sponges to inhibit their regulation of downstream cancer target genes. CircCDR1as and circMTO1, for example, bind to miR-7 and miR-9, respectively, and influence gene regulation, thus indirectly achieving either tumor inhibition or stimulation [10,11]. Due to the observed associations between circRNA abundance and cancer, circRNAs may serve as cancer biomarkers with good diagnostic performance [12].

Various studies also demonstrated that circRNAs are present at relatively high steady state levels in human biofluids, such as saliva, plasma, serum and in exosomes, which makes them attractive candidate biomarkers for noninvasive liquid biopsies [1]. For example, circ-ZEB1.33 was overexpressed in hepatocellular carcinoma (HCC) compared to adjacent normal tissue and normal liver. In line with this, the serum level of circ-ZEB1.33 was higher in HCC patients compared to healthy controls, and its levels in HCC tissue and serum were correlated across different TNM stages (TNM Classification of Malignant Tumors) and were associated with overall survival in HCC patients [13].

Numerous bioinformatics pipelines have been developed to identify circRNAs [14–17], leading to the prediction of millions of circRNAs in different short read RNA-sequencing (RNA-seq) datasets [2,3,18,19]. This spurred in the development of more than 20 databases containing human circRNAs. These databases also contain various circRNA annotations, such as circRNA tissue- and disease-specificity, circRNA–miRNA interactions, circRNA–RBP interactions, circRNA coding potential and conservation amongst species. Each has its unique aspects and merits, but we are far off a uniform consensus circRNA catalog. In this review, we present a comprehensive overview of the current circRNA databases and their content, features and usability. Furthermore, we discuss the current issues regarding circRNA databases and come with important suggestions to streamline further research in this growing field.

## Material and methods

### Literature search

PubMed and Google were queried with the following keywords: ‘circRNA database’, and all relevant hits were inspected manually. To keep the focus of our analyses on circRNA databases, only databases specific for circRNAs were included in the result tables. Other databases with interesting features are mentioned in the text. Databases exclusively containing plant circRNAs [20] were not included in this review.

### Data acquisition

All circRNA database websites were visited on 03 September 2019 using Google Chrome, Firefox and Safari.

When available, database exports were downloaded.

### Data processing

Database content was processed in RStudio (v1.2.1335). All databases containing circRNA coordinates based on the hg38 genome build were converted to hg19 using LiftOver (UCSC Genome Browser [21]). We noticed that the start positions in the files obtained from circAtlas v2.0 were one nucleotide off compared to the other databases. To compensate for this issue, the start of each BSJ in circAtlas v2.0 was lowered by one nucleotide.

The number of unique circRNAs in each database was calculated based on the BSJ or based on the unique name for the noncurated and curated databases, respectively.

Euler plots were generated with CRAN package Eulerr (v5.1.0). It is important to note that while Euler plots are a very helpful vizualisation, there is always some error, and the higher the number of diagrams, the higher the error. To ensure correct interpretation of the plots, all Euler plot results are also reported. The overlap between circRNA databases was calculated based on the BSJ position. As not all databases report the strand from which the circRNA originates, circRNAs were compared solely on their BSJ position.

The number of single-exon circRNAs was calculated by comparing the BSJ positions with all exon positions (downloaded from Ensembl, GRCh37 archive [22]).

## Results

### Overview of human circRNA databases

In total, we selected 20 human circRNAs databases and divided them into two categories: noncurated databases, based on in-house or publicly available RNA-seq or circRNA datasets; and curated databases, based on literature searches for empirically validated circRNA (Tables 1 and 2; Supplemental Table 1). Despite several attempts, Circ2Traits, CircInteractome, CircNet and deepBase v2.0 were unreachable, and therefore not included in some of our analyses. In addition, CircR2Disease and circRNADb were often found to be unreachable.

**Table 1:** Characteristics of noncurated circRNA databases. (**A**) Noncurated databases that were online accessible (**B**) Noncurated databases that were not online accessible

Database	Dataset	# circRNAs^*^	Detection tool(s)	Filter	Short description
**A**					
circAtlas v2.0 [23]	In-house data	610,406	CIRI2, DCC, MapSplice, CircExplorer2	≥2 tools ≥2 BSJ reads	Detection of circRNAs in ﻿17 human tissues reports conservation across species, circRNA-microRNA interaction, predicted ORF, IRES and RBP sites
circbank [24]	Public circRNA dataset (circBase)	140,725	Does not apply	Unknown	Focus on nomenclature system; reports miRNAs binding sites, conservation across species, m6A modification, mutations in circRNA, predicted ORFs and IRES sites
circBase [25]	Public circRNA datasets	92,375	Depends on source	None	Unified database of all circRNAs predicted by nine large-scale studies; reports putative spliced circRNA sequences
CIRCpedia v2 [26]	Public RNA-seq datasets	177,456	CIRCexplorer2, MapSplice	Unknown	Focus on circRNAs in 65 human cell lines
CircRiC [5]	Pubic RNA-seq datasets	92,599	CIRI2, find_circ, CIRCexplorer2, circRNA_finder	≥2 tools ≥2BSJ reads	Focus on detection of lineage-specific circRNAs in 935 cancer cell lines; reports drug response, biogenesis, interactions between circRNAs and mRNA (including miRNAs), proteins, or mutations
circRNADb [27]	Public circRNA datasets	32,914	Depends on source	≥2 BSJ reads	Focus on protein-coding annotation (predicted ORFs with protein-coding potential and evidence by mass spectrometry; reports exon splicing information and predicted IRE sites
CSCD [11]	Public RNA-seq datasets	1,223,114	CIRI2, find_circ, circRNA_finder, CIRCexplorer	None	Focus on distinguishing cancer-specific circRNAs from ‘normal’ circRNAs in 87 cancer cell lines and 141 normal cell lines; reports circRNA-miRNA interactions, putative splicing possibilities, predicted cellular location, RBP sites and ORFs
exoRBase [28]	Public blood exosomal RNA-seq datasets	57,412	ACFS, find_circ	≥2 tools	Focus on circRNA, IncRNA and mRNA in human blood exosomes
MiOncoCirc v2.0 [8]	In-house data	227,056	CIRCexplorer	≥2 BSJ reads	circRNA detection in 2093 clinical human cancer samples using exome capture sequencing
TSCD [29]	Public RNA-seq datasets	284,296	CIRI, find_circ, circRNAfinder	≥2 BSJ reads	Tissue-specific circRNAs in 16 adult human tissues and 15 fetal human tissues; reports circRNA-miRNA interactions, conservation across species and predicted RBP sites
**B**					
Circ2Traits [30]	Public circRNA dataset	1953^**^	find_circ	≥2 BSJ reads	Association of circRNAs with diseases based on the interactions of circRNAs with disease-associated miRNAs and disease-associated SNPs mapped on circRNA loci
CircInteractome [31]	Public circRNA dataset (circBase)	Unknown	Does not apply	Unknown	Focus on interaction between miRNAs and circRNAs with RBP sites; reports IRES sites and ORFs
CircNet [32]	Public RNA-seq datasets	34,000^**^	find_circ	≥3 sources	Maps circRNA–miRNA–mRNA interactions into regulatory networks
deepBase v2.0 [33]	Public RNA-seq and circRNA datasets (circBase)	14,867^**^	find_circ	Unknown	Comparison of (small) noncoding RNAs (including circRNAs) across 19 species; reports conservation between species

^*^Number of unique human circRNAs in the download files.

^**^Number of human circRNAs reported by the authors; this could not be verified as the database was not online.

**Table 2:** Characteristics of curated circRNA databases

Database	# circRNAs^*^	Validation method^**^	Articles prior to	Short description
Circ2Disease [34]	249	RT-(q)PCR microarray	01 November 2017	Reports curated circRNA-disease and circRNA-miRNA association
Circad [35]	925	Unknown	Unknown	Reports curated circRNA-disease associations, including detailed experimental validation
CircFunBase [36]	3181	RT-(q)PCR microarray RNA-seq	01 May 2018	Reports circRNA-associated miRNAs and circRNA-associated RBPs (CircInteractome) in seven plants and eight animals, including humans
CircR2disease [37]	599	RT-qPCR microarray	31 March 2018	Reports curated circRNA-disease associations
CircRNADisease [38]	328	RT-qPCR microarray RNA-seq	01 November 2017	Reports curated circRNA-disease associations
LncRNADisease 2.0 [39]	736	RT-(q)PCR microarray	31 May 2018	Reports curated circRNA-disease associations

^*^Number of unique human circRNAs in the download files.

^**^Most circRNAs in the database are validated by at least one of these methods. Some rarely used methods were omitted for clarity.

All curated circRNA databases employ the same content search strategy, namely a literature search with keywords such as ‘circular RNA’ and ‘circRNA disease’, followed by manual selection of suitable articles. Interestingly, apart from circRNA validation, Circ2Disease also includes manually curated circRNA–miRNAs interactions, circRNA–RBP interactions and other up- or down-stream regulatory genes.

**Figure 1:**
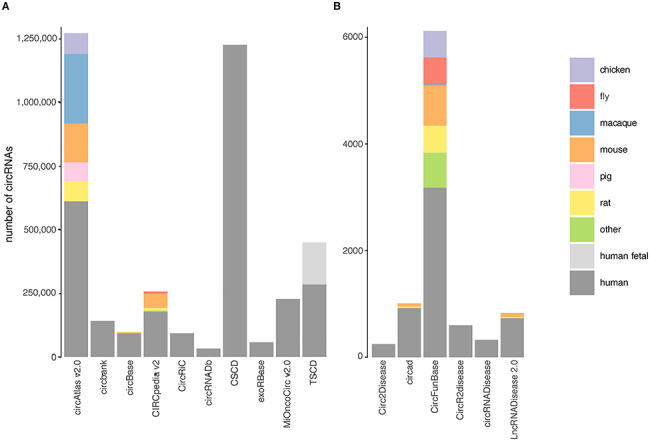
There is substantial variation in the number of circRNAs across databases. Number of unique circRNAs in the (**A**) noncurated and (**B**) curated databases. Most databases solely contain human circRNAs, but some also include circRNAs detected in other species. The number of circRNAs differs greatly between and within noncurated and curated databases. The differences within noncurated circRNA databases can mostly be explained by different starting material, sequencing method, circRNA detection tools and filtering steps. The differences within curated databases can partly be explained by different selection criteria of empirically validated circRNAs, despite similar search strategies.

While most circRNA databases predominantly store human circRNAs, a few databases also include other species such as fly, worm and mouse (Figure 1). TSCD claims to contain circRNAs detected in macaque samples but these data are currently not present in the database. Interestingly, the number of circRNAs varies substantially across the databases. In addition, each circRNA database provides different types of circRNA annotations, which are described in the following paragraphs.

### CircRNA annotation

To facilitate functional exploration of circRNAs, circRNA databases typically include several annotation levels. This section contains a short description of these annotations; a complete overview of circRNA annotations can be found in Supplemental Table 1.

#### Tissue-specificity

CircRNAs are generally annotated as tissue-specific if they are detected in specific tissues or cell types, sometimes assessed by a specificity score. CircAtlas v2.0, CIRCpedia v2 and TCSD all report circRNA expression levels across various human tissues and cell lines. In addition, CircRiC focuses on circRNAs in cancer cell lines and MiOncoCirc v2.0 on circRNAs in clinical human cancer samples.

#### CircRNA-disease associations

Due to their potential use as biomarkers, there has also been increasing interest in the association of circRNAs with diseases. These associations are mostly reported by curated databases, where circRNAs are considered disease-specific when up- or downregulated in a particular disease sample. circRNADb is a noncurated circRNA database that also reports circRNA-disease associations if there is a link between the parental gene of the circRNA and a specific disease.

#### CircRNA–miRNA interactions

In total, 12 of the databases discussed in this review (60%) report circRNA–miRNA interactions. To predict miRNA binding sites in circRNA sequences, MiRanda [40] and/or TargetScan [41] are often used. CircAtlas v2.0, circBase and circRNADisease also provide circRNA–miRNA interactions, without mentioning which miRNA database was used or how these interactions were predicted.

#### CircRNA–RBP interactions

In total, six circRNA databases (35%) report circRNA–RBP interactions. CircInteractome, CircRiC, CSCD and TSCD predict circRNA–RBP interactions based on crosslinking immunoprecipitation sequencing (CLIP-seq) data from starBase v2.0 [42]. The remaining two, CircFunBase and Circ2Disease, use the predicted RBP–circRNA interactions from CircInteractome (unfortunately offline).

#### Protein-coding potential

Although circRNAs are generally classified as noncoding RNAs, eukaryotic ribosomes can initiate translation of engineered circRNA when containing an internal ribosome entry site (IRES) element [26]. Furthermore, several human circRNAs are shown to be translated *in vivo* [43]. Therefore, some databases report predicted IRES or predicted open reading frames (ORFs). circRNADb provides the richest information on the protein-coding potential of circRNAs. It contains predicted IRES elements in the spliced sequence of each circRNA using VIPS (viral IRES prediction system) [44], and it also predicts the longest potential ORF. Other circRNA databases rely on CPAT (coding-potential assessment tool) [45] or ORF Finder (from NCBI), and IRESfinder [46] or IRESite [47] to predict the coding potential and IRES elements, respectively.

#### CircRNA conservation

As conservation of a particular genomic sequence may hint at a functional role, multiple researchers have investigated the conservation of circRNAs. CircAtlas v2.0, circbank and CIRCpedia v2 classify circRNAs from different species as orthologs when the BSJ site is conserved within a small 2–5 nucleotide range.

#### Other annotations

Finally, some circRNA databases have unique annotation features. For example, CircRiC reports the correlation between host gene expression and normalized BSJ read numbers. Furthermore, CircRiC also includes associations between drug response and the expression of circRNAs. MiOncoCirc v2.0 developed a pipeline (CODAC) to identify back-splicing involving two genes. Additionally, multiple databases report putative circRNA functions based on gene ontology enrichment analysis.

## Discussion

### There is little overlap among public circRNA databases

**Figure 2:**
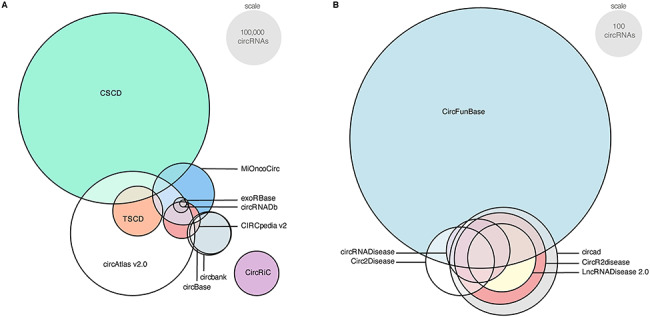
Varying degrees of overlap among circRNA databases. To assess the content of circRNA databases, the overlap within (**A**) noncurated and within (**B**) curated databases was calculated. First, all non-hg19-based databases were converted to hg19 using LiftOver (UCSC Genome Browers [21]), and subsequently a Euler plot was computed. Of note, while an Euler plot is helpful for vizualisation, it is not entirely accurate and the plotted overlap is the approximation with the smallest error. For example, 35% of circRNAs present in CircRiC are also present in at least one of the other noncurated databases, however it was not possible to show this in the Euler plot. The exact overlap between all circRNA databases can be consulted in Supplemental Table 2.

In total, 20 databases were included in this review, of which 14 noncurated and six curated. To assess the overlap among the circRNA databases, Euler plots were generated (Figure 2 and Supplemental Table 2).

The noncurated databases (Figure 2A) were divided into two groups, either based on *de novo* generated circRNA data or based on publicly available circRNA datasets (distinction also indicated in Table 1). First, for the databases that use publicly available circRNA datasets (circbank, circBase, circRNADb, CircInteractome, deepBase v2.0), we expect to see a high degree of overlap, as they often reuse the same datasets. While circbank, CircInteractome and deepBase v2.0 all use the circBase circRNA dataset as input, the contents of CircInteractome and deepBase v2.0 could not be assessed, as their databases were not online. Furthermore, although circBase completely overlaps with circbank, somehow circbank contains more circRNAs than circBase, an observation we were unable to find an explanation for. circBase itself is based on nine circRNA datasets, including Jeck *et al*. [18] and Memczak *et al*. [19], two circRNA datasets that are also included in circRNADb (based on four datasets in total). Next, for the databases that use in-house or publicly available RNA-seq data (circAtlas v2.0, CIRCpedia v2, CircRiC, CSCD, exoRBase, MiOncoCirc v2.0 and TSCD), we observe little overlap. This is not unexpected as these databases rely on different samples, and circRNAs are expressed at low levels and with high sample-specificity [3,5]. Additionally, these circRNA databases applied different sequencing methods (varying RNA input levels, library preparation, sequencing dept, all affecting the sensitivity of the circRNA detection), circRNA detection tools and filtering steps, further contributing to the difference in content between the noncurated circRNA databases. The overlap between noncurated databases increases when filtered for experimentally validated circRNAs (circRNAs present in at least one curated database), as this also increases the probability of true positive circRNAs (Supplemental Figure 1).

It is thus extremely important to consider what samples were used to build the circRNA database and select a database in line with the tissue of interest. Moreover, the detection of circRNA in RNA-seq data does not guarantee that the predicted circRNAs are true positives. Therefore, some databases allow filtering for circRNAs detected by at least two tools, which improves the reliability of the predictions [48].

Overall, noncurated circRNA databases seem to contain a high number of circRNAs, whereby the reliability of these circRNAs must be questioned, as no validation using an orthogonal method is reported. Besides, it is important to recognize that circRNA expression and detection can vary considerably depending on multiple factors such as sample type, sequencing method and circRNA detection tool.

Six databases were found containing curated circRNA disease or function associations. Combined, these databases add up to 3522 circRNAs that have been empirically validated to date (Supplemental Table 3). Despite similar search strategies, there are notable differences in the content of curated databases (Figure 2B). Of note, not all databases apply the same criteria to label circRNAs as empirically validated. For the unpublished database circad, there is no information available on the accepted validation methods. Overall, the curated databases accept circRNAs validated by reverse transcriptase (quantitative) polymerase chain reaction (RT-(q)PCR), microarray or northern blot. However, CircFunBase and circRNADisease also accept RNA-seq as a sufficient method of circRNA validation. Seven hundred and forty four out of 3181 (23%) and 17 out of 328 (5%) circRNAs were solely detected by RNA-seq, respectively. It is not reported if a circRNA enrichment step (e.g. using RNAse R) was used as part of the RNA-seq validation, moreover both RNA-seq with and without circRNA enrichment can be found in the validated circRNAs when manually inspected. As a universal method for circRNA validation is lacking, we urge researchers to be more cautious and rely on multiple detection methods for effective validation of circRNAs.

Another explanation for the limited overlap between curated databases could be the redundancy arising from misannotated chromosomal positions. Usually, coordinates (chr:start-end) are given with inclusive start and end position. However, some formats (such as Browser Extensible Data, BED format) use a 0-based exclusive start position. Uncareful curation of circRNA positions from literature could thus result in incorrect or even redundant annotation. For example, there are two nearly identical CDR1 circRNAs present in CircFunBase, one with position chrX:139865340-139866824, and the other one with position chrX:139865339-139866824 (both hg19). The former is only supported by one publication, in contrast to the latter, which is supported by multiple publications and is also present in other databases (hsa_circ_0001946). However, these are probably the same molecules with BSJ positions based on different annotation systems.

In total, there are more than 2 million different circRNAs present in the union of all noncurated databases (compared to 384,066 predicted human RNA transcripts [49]), and 3522 circRNAs in the union of all curated databases. Surprisingly, more than 500 curated circRNAs are not present in any of the noncurated databases. This can partly be explained by misannotated start positions, as was previously discussed for CircFunBase. Although this issue does not completely explain these 500 circRNAs solely detected in curated databases, we expect that the remaining loss in overlap is due to other annotation related-issues. It is therefore recommended, when comparing datasets, to ensure that the annotations are compatible, or adjust them if necessary.

Further illustration of the lack in overlap can be seen in Supplemental Figure 2, which shows that most circRNAs are only present in one database.

### The full-length sequence of circRNAs is lacking

Multiple databases (30%) provide full-length circRNA sequences and use it to predict interactions with miRNA and other sequence-based interaction partners. Unfortunately, the full-length sequence of most circRNAs is not known to date as full-length length circRNA sequencing datasets are lacking. Rather, the sequence between the start and stop position of the circRNA is inferred based on the reference genome sequence. The databases seem to report full-length circRNA sequences based on all known exons from the linear transcript. Most databases remove the introns, with the exception of circAtlas v2.0, which reports circRNA sequences based on exons and introns. This, however, relies on the unsupported assumption that circular and linear transcripts share the same splicing pattern and RNA sequence. However, almost 50% of the circRNA host genes give rise to multiple (up to 20) circular isoforms each [50]. Additionally, the inferred circRNA sequence depends on the genome build and on the exon and transcript annotation. CircFunBase reports multiple full-length circRNA sequences based on all overlapping linear transcripts reported by Ensembl, without taking into account alternative splicing.

In fact, all the databases included in this review should rather be called BSJ databases instead of circRNA databases, as none of them provide the empirically validated full-length sequence of circRNAs. An exception could be made for single-exon circRNAs, as it can be assumed that their sequence is the same as their parental linear exons. Interestingly, only 1.5% of circRNAs from the selected databases seem to be single-exon circRNAs. Whether this is caused by annotation problems in the different databases or is a true feature of circRNA biogenesis is unclear at this point.

**Table 3:** Ambiguous circRNA nomenclature contributes to the reproducibility crisis. Listed here are the different naming systems used across circRNA databases. While some naming systems are very similar, the differences are subtle and can easily lead to mistakes. The number used as index in the table indicates the number of digits used for the circRNA name in the database

Database	circGENE	hsa_circ_000006	hsa_circ_0000007	hsa_circRNA_000006	hsa_circRNA_0000007	chr:start|end	Unique nomenclature system*
Circ2Disease	x	x	x				circR-003
circad	x	x	x	x	x		
circAtlas v2.0							hsa-GENE_000006
circbank			x				hsa_circGENE_003
circBase			x				
CircFunBase		x	x				
CIRCpedia v2							HSA_CIRCpedia_00005
CircR2Disease	x	x	x	x	x		circRNA_000006
CircRiC							chr_start_end_GENE
circRNADb							hsa_circ_00005
circRNADisease	x	x	x				
CSCD						x	
exoRBase			x				exo_circ_000006
LncRNADisease 2.0	x						
MiOncoCirc v2.0							chr:start:end:GENE-AS
TSCD						x	

^*^This nomenclature system is used by a single database.

While some tools, including CIRI-full [51] and circseq_cup [52], were developed to detect full-length circRNA sequences based on RNA-seq data, current databases do not make use of this type of analysis. CIRI-full makes use of a novel feature called reverse overlap and of the BSJ sites to reconstruct full-length circRNAs and circular isoforms. Circseq_cup first identifies BSJ sites and then assembles the full-length sequences of circRNAs using the paired-end reads aligned to the BSJ. Apart from computational methods, full-length circRNAs have been unambiguously identified using long-read single-molecule sequencing [53] or rolling circle amplification in combination with Sanger sequencing [54].

Another important characteristic of a circRNA is the DNA strand from which it is transcribed. Six of the databases we reviewed (30%) do not report the originating strand. Moreover, circRNADb does not report the gene nor the strand, but a link is provided to circBase, where the strand can be found. If the host gene is not mentioned, it is crucial to mention the strand from which the circRNA is transcribed (and hence stranded RNA sequencing methods should be used) to be able to identify the circRNA correctly.

### Ambiguous circRNA nomenclature contributes to the reproducibility crisis

Until now, no consensus circRNA nomenclature has been established. As indicated in Table 3, several similar nomenclature systems are in use, leading to multiple issues and increasing the risk of mistakes and confusion. The various nomenclature systems differ slightly in their prefix and the number of digits in the index. Next to the same circRNA having multiple names, nearly identical names with the same index sometimes correspond to different circRNAs. We illustrate this issue using circMTO1, which has at least 11 different names (Table 4). CircMTO1 (hsa_circ_0007874, hg19: chr6:74175931–74,176,329) is a circRNA that acts as a miRNA sponge for multiple RNA molecules, including oncogenic miR-9 [11] and is linked to HCC [55]. circMTO1 is referred to as hsa_circ_30012 in circRNADb and hsa_circ_0007874 in circBase. It is problematic that hsa_circ_0030012 is a completely different circRNA in circBase, transcribed from FAM48A (hg19: chr13:37598171-37625720) and hsa_circ_07874 in circRNADb is also a completely different molecule (hg19: chr2:55040368-55047599). Similarly, while hsa-MTO1_000001 corresponds to a molecule with a BSJ at position chr6:74175932-74202075 in circAtlas v2.0, circbank gives other BSJ coordinates to hsa_circMTO1_001, namely chr6:74175931-74176329 (matching the position mentioned in circBase). To further illustrate this issue, a second example presenting a list of all 13 names given to ciRS-7 (hg19: X_139865339:139866824) can be found in Supplemental Table 4.

**Table 4:** Eleven different names of circMTO1 illustrate the reproducibility crisis caused by ambiguous circRNA nomenclature. CircMTO1 is a well-studied circRNA positioned at chr6:74175931–74,176,329 (hg19). Despite several publications on circMTO1, the circRNA does not have a universal name in the circRNA databases and is referred to by eleven different identifiers

Name for circMTO1	Used in database
circMTO1	Circ2Disease, circad, CircR2Disease, circRNADisease, LncRNADisease 2.0
hsa_circ_0007874	Circ2Disease, circad, circbank, circBase, CircFunBase, circRNADisease, exoRBase
hsa_circRNA_104135	Circ2Disease, circad, CircR2Disease
hsa_circ_104135	circad, circRNADisease
hsa_circRNA_0007874	circad, CircR2Disease
hsa_circMTO1_001	circbank
HSA_CIRCpedia_55478	CIRCpedia v2
6_74,175,931_74,176,329_MTO1	CircRiC
hsa_circ_30012	circRNADb
exo_circ_006565	exoRBase
chr6:73466208:73466606:MTO1	MiOncoCirc v2.0

Fortunately, most circRNA databases report the circRNA alias used in circBase, which was one of the first large circRNA databases. This is with the exception of circAtlas v2.0, CIRCpedia v2, CircRiC, circRNADb, CSCD and MiOncoCirc v2.0. The only way to compare the content of these databases is by using the BSJ position, which is not convenient.

Until now, the circBase nomenclature (hsa_circ_0000007) seems to be the most widely used naming system. However, it would be useful to work with a nomenclature that includes the host gene name, such as circbank proposes. This makes the name more human-readable and can prevent mistakes. We recommend combining the species (e.g. hsa for human), the nature of the RNA molecule (circ), the official gene symbol (GENE), a then a unique identifier for each circRNA for that gene. While this unique identifier could be a simple three-digit number (from one to the total number of circRNAs identified for that gene), it would be more informative to have two indices (e.g. one to indicate the position of the BSJ and the second one to indicate the splicing pattern, once the full-length sequence of that specific circRNA is known). For example, two circRNAs from the same gene with the same BSJ, but with a different internal sequence, could be called hsa_circGENE_001_001 and hsa_circGENE_001_002. CircRNAs from which the full-length sequence is unknown, could be indicated by the _000 suffix. Alternatively, the strategy currently used by the miRNA database miRbase [56] can be applied, where instead of a second index a letter is used to indicate the full-length sequence. For example: hsa_circGENE_001a for the first known sequence and hsa_circGENE_001 for the unknown sequence. Using the gene symbol of the host gene poses another important issue: the naming system cannot be applied when the host gene lacks an official symbol, as is the case for many long noncoding RNAs (lncRNAs). Another unique identifier could be a hash that represents the full-length circRNA sequence or the 25 nucleotides flanking the BSJ if the full-length sequence is unknown. In any case, if a naming system without a host gene is used, it is crucial to report the strand from which the circRNA is transcribed, otherwise, the name can again refer to different circRNAs.

### CircRNA annotation is mostly based on assumptions and predictions

Almost all circRNA databases report at least one type of circRNA annotation (*vide supra*), but these annotations should be handled with care. First, circRNA annotations are mostly based on computational predictions rather than experimental validation. Second, some sequence-based annotations, such as miRNA and RBP binding are predicted based on the presumed full-length sequence of circRNA molecules. As stated before, the empirically validated full-length sequence of a circRNA is generally lacking, and it is often not mentioned if the circRNA sequence used for prediction is the full-length sequence based on the reference genome, with or without taking splicing into account, or if the sequence is based on RNA-seq reads containing the BSJ. Third, it is important to note that the mere detection of a circRNA in a specific tissue or cell type does not necessarily indicate that this circRNA is specifically expressed. This also goes for curated circRNA databases, where the up- or downregulation of a circRNA in a specific disease sample in comparison with a control is often used to label a circRNA disease-specific.

Finally, a lot of databases do not report the source of their annotations and predictions. Overall, we would like to warn users of circRNA databases to be aware of the limitations regarding circRNA annotations.

### Updates, user interface and availability

Although almost all authors of circRNA database articles mention the importance of regular updates and promise to maintain their database, only circBase and CIRCpedia v2 seem to have been updated after publication (in July 2017 and July 2018, respectively). Of note, some databases are very recent at the time of writing and might be updated in the near future. Unfortunately, some databases were completely inaccessible online, and some database exports were incomplete. Also, some databases are difficult to use or are limited to specific web browsers.

## Conclusions

In this review, we provide an overview of all databases focused on human circRNA, divided into noncurated and curated circRNA databases. In total, there are more than 2 million different circRNAs present in the union of all noncurated databases, and 3522 circRNAs in the union of all curated databases. Generally speaking, there is limited overlap among these databases. The lack of overlap between noncurated databases can be explained by the use of different samples and the nature of circRNAs (low abundance, high sample-specificity) on the one hand, and by varying sequencing methods, circRNA detection tools and filtering on the other hand. It is important to be aware of the sample-specificity of circRNAs, and a database should be selected with care when conducting circRNA research. The lack in overlap among the curated databases might be due to different filtering techniques when selecting literature, and due to annotation-related issues. Furthermore, the use of different nomenclature systems is leading to redundancy and could cause confusion amongst circRNA researchers. This issue may very well contribute to the reproducibility crisis, and therefore we propose clear future guidelines for a solid circRNA nomenclature. Also, it is crucial to realize that the circRNA BSJ is not a unique identifier of a specific circRNA molecule, as splicing needs to be taken into account as well. Due to the lack of full-length circRNA sequences, multiple sequence-based annotations are predicted based on the assumption that circRNAs following the same splicing pattern as their parental mRNA counterparts and are thus unreliable. Finally, several databases are not regularly updated or suffer from connectivity issues.

## Supplementary Material

supplemental_figure_1_bbz175

supplemental_figure_2_bbz175

supplemental_table_1_bbz175

supplemental_table_2_bbz175

supplemental_table_3_bbz175

supplemental_table_4_bbz175

supplementary_data_bbz175

## Notes

Key Points

- There is limited overlap among circRNA databases, a result of the different source material and the nature of circRNAs (low abundance, high sample-specificity) on the one hand, and of varying sequencing methods, circRNA detection tools and filtering on the other hand.
- The ambiguous nomenclature of circRNAs resulted in conflicting names for circRNAs in different databases, contributing to the reproducibility crisis. One uniform naming system should be carefully implemented to prevent further future confusion.
- The BSJ position on itself is not sufficient to uniquely identify a circRNA, and the full-length sequence of most circRNAs is lacking.
- Many circRNA databases report interactions with miRNA and RBPs, protein-coding potential, conservation between species, etc. These predictions should be used with caution, as they are generally based on the assumed full-length sequence of circRNAs.

## Notes

**Marieke Vromman** is a doctoral fellow at the department of Biomolecular Medicine at Ghent University and a member of the Cancer Research Institute Ghent.

## Notes

**Jo Vandesompele** is professor at the department of Biomolecular Medicine at Ghent University and a group leader at the Cancer Research Institute Ghent.

## Notes

**Pieter-Jan Volders** is a post-doctoral fellow at the department of Biomolecular Medicine at Ghent University and at the Flemish Institute for Biotechnology, and a member of the Cancer Research Institute Ghent.

## References

[1] Su M, Xiao Y, Ma J, et al. Circular RNAs in cancer: emerging functions in hallmarks, stemness, resistance and roles as potential biomarkers. Mol Cancer 2019;18:90. 30999909 10.1186/s12943-019-1002-6 PMC6471953

[2] Salzman J, Gawad C, Wang PL, et al. Circular RNAs are the predominant transcript isoform from hundreds of human genes in diverse cell types. PLoS One 2012;7:e30733. 22319583 10.1371/journal.pone.0030733 PMC3270023

[3] Salzman J, Chen RE, Olsen MN, et al. Cell-type specific features of Circular RNA expression. PLoS Genet 2013;9(1):13. 10.1371/journal.pgen.1003777 PMC3764148 24039610

[4] Li HM, Ma XL, Li HG Intriguing circles: conflicts and controversies in circular RNA research. Wiley Interdiscip. Rev. RNA 2019;10:e1538. 31034768 10.1002/wrna.1538

[5] Hang R, Xiang YU, Ko J, et al. Comprehensive characterization of circular RNAs in ~1000 human cancer cell lines. Genome Med 2019;11:55. 31446897 10.1186/s13073-019-0663-5 PMC6709551

[6] Barrett SP, Salzman J Circular RNAs: analysis, expression and potential functions. Dev 2016;143:1838–47. 10.1242/dev.128074 PMC4920157 27246710

[7] Li X, Yang L, Chen LL The biogenesis, functions, and challenges of Circular RNAs. Mol Cell 2018;71:428–42. 30057200 10.1016/j.molcel.2018.06.034

[8] Vo JN, Cieslik M, Zhang Y, et al. The landscape of Circular RNA in cancer. Cell 2019;176:869–81. 30735636 10.1016/j.cell.2018.12.021 PMC6601354

[9] Shang Q, Yang Z, Jia R, et al. The novel roles of circRNAs in human cancer. Mol Cancer 2019;18:6. 30626395 10.1186/s12943-018-0934-6 PMC6325800

[10] Wu J, Qi X, Liu L, et al. Emerging epigenetic regulation of Circular RNAs in human cancer. Mol Ther - Nucleic Acids 2019;16:589–96. 31082792 10.1016/j.omtn.2019.04.011 PMC6517616

[11] Xia S, Feng J, Chen K, et al. CSCD: a database for cancer-specific circular RNAs. Nucleic Acids Res 2018;46:D925–9. 29036403 10.1093/nar/gkx863 PMC5753219

[12] Tan H, Gan L, Fan X, et al. Diagnostic value of circular RNAs as effective biomarkers for cancer: a systematic review and meta-analysis. Onco Targets Ther 2019;12:2623–33. 31114221 10.2147/OTT.S197537 PMC6497823

[13] Gong Y, Mao J, Wu D, et al. Circ-ZEB1.33 promotes the proliferation of human HCC by sponging miR-200a-3p and upregulating CDK6. Cancer Cell Int 2018;18:116. 30123094 10.1186/s12935-018-0602-3 PMC6090603

[14] Gao Y, Zhao F Computational strategies for exploring Circular RNAs. Trends Genet 2018;34:389–400. 29338875 10.1016/j.tig.2017.12.016

[15] Hansen TB, Venø MT, Damgaard CK, et al. Comparison of circular RNA prediction tools. Nucleic Acids Res 2015;44:e58. 26657634 10.1093/nar/gkv1458 PMC4824091

[16] Zeng X, Lin W, Guo M, et al. A comprehensive overview and evaluation of circular RNA detection tools. PLoS Comput Biol 2017;13:e1005420. 28594838 10.1371/journal.pcbi.1005420 PMC5466358

[17] Jakobi T, Dieterich C Computational approaches for circular RNA analysis. Wiley Interdiscip Rev RNA 2019;1–14. 10.1002/wrna.1528 30788906

[18] Jeck W, Sorrentino J, Wang K, et al. Circular RNAs are abundant, conserved, and associated with ALU repeats. RNA 2013;19:141–57. 23249747 10.1261/rna.035667.112 PMC3543092

[19] Memczak S, Jens M, Elefsinioti A, et al. Circular RNAs are a large class of animal RNAs with regulatory potency. Nature 2013;495:333–8. 23446348 10.1038/nature11928

[20] Zhao W, Chu S, Jiao Y Present scenario of circular RNAs (circRNAs) in plants. Front Plant Sci 2019;10:379. 31001302 10.3389/fpls.2019.00379 PMC6454147

[21] Haeussler M, Zweig AS, Tyner C, et al. The UCSC genome browser database: 2019 update. Nucleic Acids Res 2019;47:D853–8. 30407534 10.1093/nar/gky1095 PMC6323953

[22] Yates AD, Achuthan P, Akanni W, et al. Ensembl 2020. Nucleic Acids Res 2019;48:D682–8. 10.1093/nar/gkz966 PMC7145704 31691826

[23] Ji P, Wu W, Chen S, et al. Expanded expression landscape and prioritization of Circular RNAs in mammals. Cell Rep 2019;26:3444–60. 30893614 10.1016/j.celrep.2019.02.078

[24] Liu M, Wang Q, Shen J, et al. Circbank: a comprehensive database for circRNA with standard nomenclature. RNA Biol 2019;16:899–905. 31023147 10.1080/15476286.2019.1600395 PMC6546381

[25] P G, P P, circBase NR A database for circular RNAs. RNA 2014;20:1666–70. 25234927 10.1261/rna.043687.113 PMC4201819

[26] Dong R, Ma XK, Li GW, et al. CIRCpedia v2: an updated database for comprehensive Circular RNA annotation and expression comparison. Genomics. Proteomics Bioinforma 2018;16:226–33. 10.1016/j.gpb.2018.08.001 PMC6203687 30172046

[27] Chen X, Han P, Zhou T, et al. CircRNADb: a comprehensive database for human circular RNAs with protein-coding annotations. Sci Rep 2016;6:34985. 27725737 10.1038/srep34985 PMC5057092

[28] Li S, Li Y, Chen B, et al. ExoRBase: a database of circRNA, lncRNA and mRNA in human blood exosomes. Nucleic Acids Res 2018;46:D106–12. 30053265 10.1093/nar/gkx891 PMC5753357

[29] Xia S, Feng J, Lei L, et al. Comprehensive characterization of tissue-specific circular RNAs in the human and mouse genomes. Brief Bioinform 2017;18:984–92. 27543790 10.1093/bib/bbw081

[30] Ghosal S, Das S, Sen R, et al. Circ2Traits: a comprehensive database for circular RNA potentially associated with disease and traits. Front Genet 2013;4:283. 24339831 10.3389/fgene.2013.00283 PMC3857533

[31] Dudekula DB, Panda AC, Grammatikakis I, et al. Circinteractome: a web tool for exploring circular RNAs and their interacting proteins and microRNAs. RNA Biol 2016;13:34–42. 26669964 10.1080/15476286.2015.1128065 PMC4829301

[32] Liu YC, Li JR, Sun CH, et al. CircNet: a database of circular RNAs derived from transcriptome sequencing data. Nucleic Acids Res 2016;44:D209–15. 26450965 10.1093/nar/gkv940 PMC4702939

[33] Zheng LL, Li JH, Wu J, et al. deepBase v2.0: identification, expression, evolution and function of small RNAs, LncRNAs and circular RNAs from deep-sequencing data. Nucleic Acids Res 2016;44:D196–202. 26590255 10.1093/nar/gkv1273 PMC4702900

[34] Yao D, Zhang L, Zheng M, et al. Circ2Disease: a manually curated database of experimentally validated circRNAs in human disease. Sci Rep 2018;8:11018. 30030469 10.1038/s41598-018-29360-3 PMC6054656

[35] Rophina M, Disha S, Mukta P, et al. Circad: a manually curated database of circular RNAs associated with diseases http://clingen.igib.res.in/circad/ (8 January 2020, date last accessed).

[36] Meng X, Hu D, Zhang P, et al. CircFunBase: A database for functional circular RNAs. Database 2019; 2019:baz003. 10.1093/database/baz003 PMC6360206 30715276

[37] Fan C, Lei X, Fang Z, et al. CircR2Disease: a manually curated database for experimentally supported circular RNAs associated with various diseases. Database 2018;2018:bay044. 10.1093/database/bay044 PMC5941138 29741596

[38] Zhao Z, Wang K, Wu F, et al. CircRNA disease: a manually curated database of experimentally supported circRNA-disease associations. Cell Death Dis 2018;9:475. 29700306 10.1038/s41419-018-0503-3 PMC5919922

[39] Bao Z, Yang Z, Huang Z, et al. LncRNADisease 2.0: an updated database of long non-coding RNA-associated diseases. Nucleic Acids Res 2019;47:D1034–7. 30285109 10.1093/nar/gky905 PMC6324086

[40] John B, Enright AJ, Aravin A, et al. Human microRNA targets. PLoS Biol 2004;2:e363. 15502875 10.1371/journal.pbio.0020363 PMC521178

[41] Grimson A, Farh KKH, Johnston WK, et al. MicroRNA targeting specificity in mammals: determinants beyond seed pairing. Mol Cell 2007;27:91–105. 17612493 10.1016/j.molcel.2007.06.017 PMC3800283

[42] Li JH, Liu S, Zhou H, et al. StarBase v2.0: decoding miRNA-ceRNA, miRNA-ncRNA and protein-RNA interaction networks from large-scale CLIP-Seq data. Nucleic Acids Res 2014;42:92–7. 10.1093/nar/gkt1248 PMC3964941 24297251

[43] Schneider T, Bindereif A, Circular RNA Coding or noncoding? Cell Res 2017;27:724–5. 28508860 10.1038/cr.2017.70 PMC5518877

[44] Hong JJ, Wu TY, Chang TY, et al. Viral IRES prediction system - a web server for prediction of the IRES secondary structure in Silico. PLoS One 2013;8:e79288. 24223923 10.1371/journal.pone.0079288 PMC3818432

[45] Wang L, Park HJ, Dasari S, et al. CPAT: coding-potential assessment tool using an alignment-free logistic regression model. Nucleic Acids Res 2013;41:e74–4. 23335781 10.1093/nar/gkt006 PMC3616698

[46] Zhao J, Wu J, Xu T, et al. IRESfinder: identifying RNA internal ribosome entry site in eukaryotic cell using framed k-mer features. J Genet Genomics 2018;45:403–6. 30054216 10.1016/j.jgg.2018.07.006

[47] Mokrejš M, Mašek T, Vopálenský V, et al. IRESite a tool for the examination of viral and cellular internal ribosome entry sites. Nucleic Acids Res 2009;38:D131–6. 19917642 10.1093/nar/gkp981 PMC2808886

[48] Hansen TB Improved circRNA identification by combining prediction algorithms. Front Cell Dev Biol 2018;6:20. 29556495 10.3389/fcell.2018.00020 PMC5844931

[49] Iyer MK, Niknafs YS, Malik R, et al. The landscape of long noncoding RNAs in the human transcriptome. Nat Genet 2015;47:199–208. 25599403 10.1038/ng.3192 PMC4417758

[50] Gaffo E, Boldrin E, Dal Molin A, et al. Circular RNA differential expression in blood cell populations and exploration of circRNA deregulation in pediatric acute lymphoblastic leukemia. Sci. Rep 2019;9:14670. 10.1038/s41598-019-50864-z PMC6789028 31605010

[51] Zheng Y, Ji P, Chen S, et al. Reconstruction of full-length circular RNAs enables isoform-level quantification. Genome Med 2019;11:2. 30660194 10.1186/s13073-019-0614-1 PMC6339429

[52] Guo L, Zhu Q-H, Zhang X, et al. Full-length sequence assembly reveals circular RNAs with diverse non-GT/AG splicing signals in rice. RNA Biol 2016;14:1055–63. 27739910 10.1080/15476286.2016.1245268 PMC5680721

[53] Rahimi K, Venø MT, Dupont DM, et al. Nanopore sequencing of full-length circRNAs in human and mouse brains reveals circRNA-specific exon usage and intron retention. bioRxiv 2019;567164. 10.1038/s41467-021-24975-z PMC8355340 34376658

[54] Das A, Rout PK, Gorospe M, et al. Rolling circle cDNA synthesis uncovers Circular RNA splice variants. Int J Mol Sci 2019;20:3988. 10.3390/ijms20163988 PMC6721031 31426285

[55] Han D, Li J, Wang H, et al. Circular RNA circMTO1 acts as the sponge of microRNA-9 to suppress hepatocellular carcinoma progression. Hepatology 2017;66:1151–64. 28520103 10.1002/hep.29270

[56] G-JS miRBase: microRNA sequences. targets and gene nomenclature Nucleic Acids Res 2006;34:D140–4. 16381832 10.1093/nar/gkj112 PMC1347474

