# Supplementary figures and images for: Closing the circle: current state and perspectives of circular RNA databases
Source: Brief Bioinform. 2020 Jan 30;22(1):288–97. doi: 10.1093/bib/bbz175 (PMC7820840; doi:10.1093/bib/bbz175)

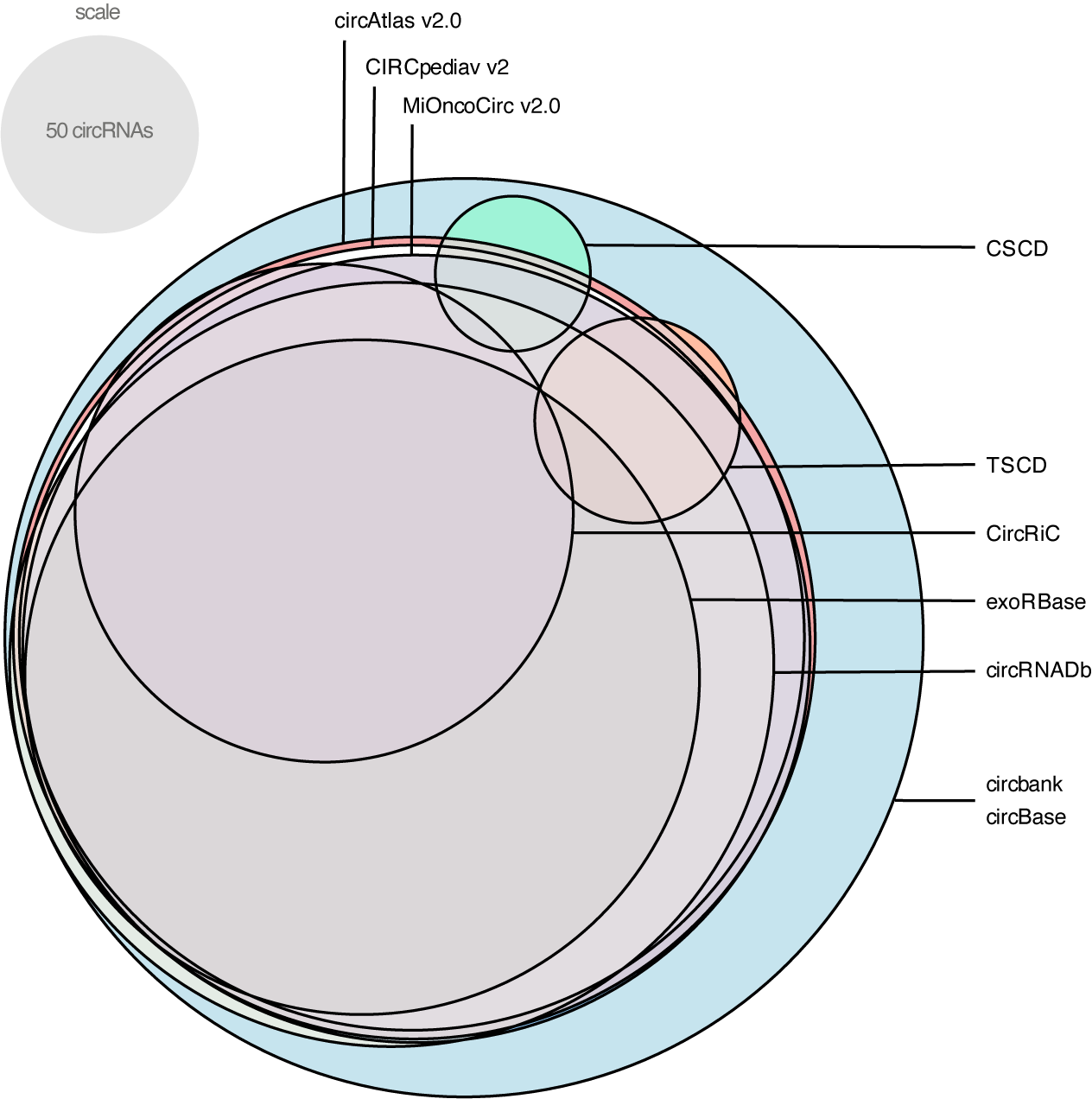

Supplement: supplemental_figure_1_bbz175 [file supplemental_figure_1_bbz175.png]

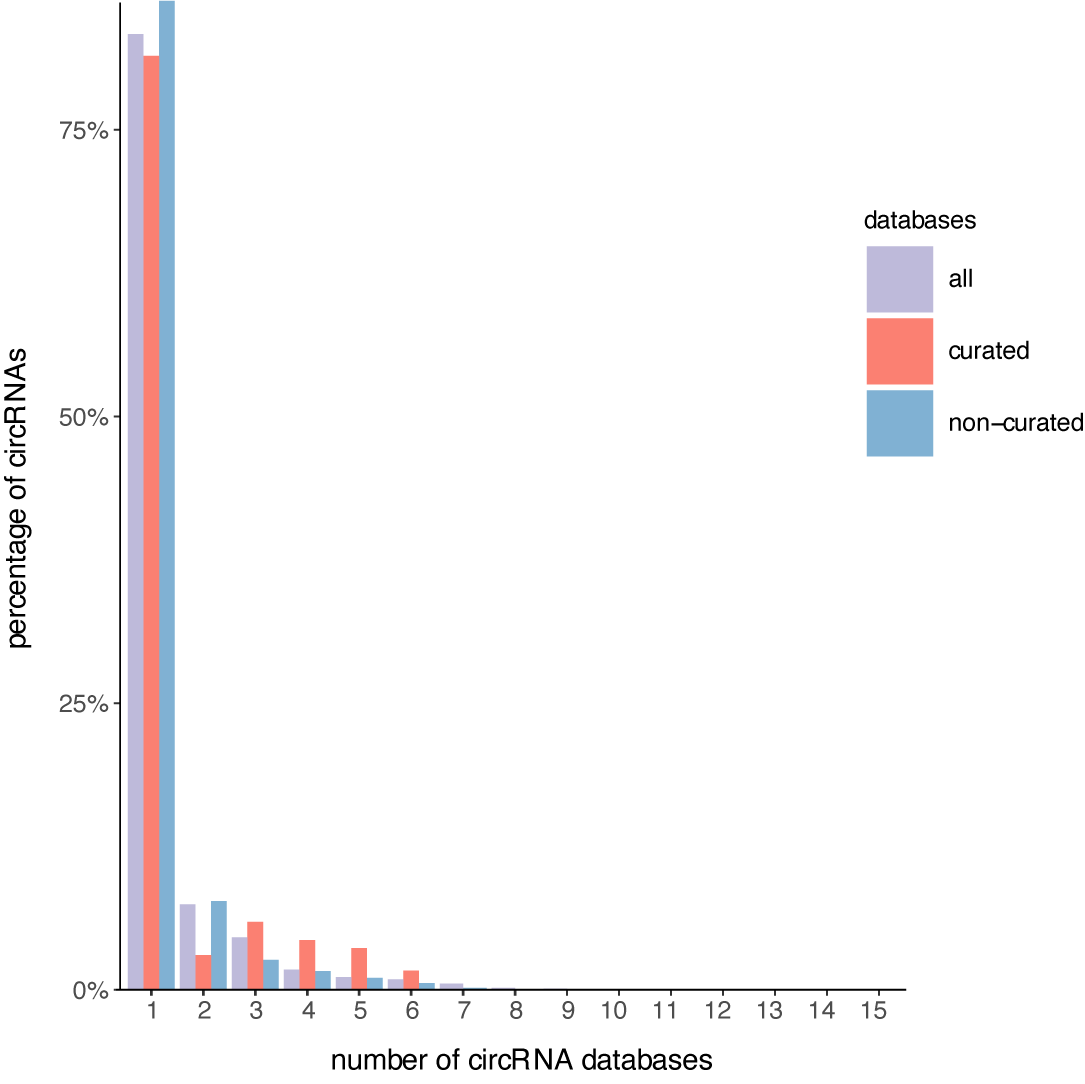

Supplement: supplemental_figure_2_bbz175 [file supplemental_figure_2_bbz175.png]
