# Supplementary material for: Closing the circle: current state and perspectives of circular RNA databases
Source: Brief Bioinform. 2020 Jan 30;22(1):288–97. doi: 10.1093/bib/bbz175 (PMC7820840; doi:10.1093/bib/bbz175)
Supplement: supplementary_data_bbz175 [file supplementary_data_bbz175.docx]

**Supplementary Data**

Two supplementary figures and four supplementary tables are available as supplementary data.

**Supplemental Figure 1 The overlap between non-curated databases increases when filtered for experimentally validated circRNAs.** All non-curated databases were filtered for circRNAs present in at least one curated database. This increases the probability of true positive circRNAs, and therefore also increases the amount of overlap between the non-curated databases.

**Supplemental Figure 2 Most circRNAs are only present in one circRNA database.** Shown here is the amount of circRNAs that are present in one or multiple circRNA databases. Most circRNAs are only present in one database, almost independent of their curation state.

**Supplemental Table 1** All circRNA data as used to construct the Euler plots (Figure 2, Supplemental Figure 1).

**Supplemental Table 2** Detailed overview of all circRNA databases included in this review.

**Supplemental Table 3** List of all curated circRNAs and in which curated databases they appear. The last column indicates if the circRNA is also present in at least one of the non-curated databases.

**Supplemental Table 4 Thirteen different names of ciRS-7 illustrate the reproducibility crisis caused by ambiguous circRNA nomenclature.** ciRS-7is a well-studied circRNA positioned at X_139865339:139866824 (hg19). Despite several publications on ciRS-7, the circRNA does not have a universal name in the circRNA databases and is referred to by eleven thirteen identifiers.
